# Democratising complex system modelling

**DOI:** 10.1038/s41598-024-61318-6

**Published:** 2024-05-08

**Authors:** Anabele-Linda Pardi, Elizaveta Burina

**Affiliations:** 1 HafenCity University, 20457 Hamburg, Germany; 2 Centre d’Économie de la Sorbonne, Université Paris 1 Panthéon-Sorbonne, 75005 Paris, France

**Keywords:** Chemistry, Stability analysis, Complex systems, Applied mathematics, Reaction mechanisms

## Abstract

In the contemporary context of an acute need for sustainability and swift response to imminent crises such as global warming, pandemics and economic system disruptions, the focus on responsible decision making, ethical risk assessment and mitigation at all organizational levels is an overarching goal. Our aim is to introduce a deterministic method for investigating the stability of complex systems, in order to find the most important elements of such systems and their impact on different scenarios. The novelty of the current approach lies in its compact format and intuitive nature, designed to accommodate a limited amount of computational resources. The proposed modelling method involves the mapping of complex systems from a diversity of disciplines (economic markets, resource management domain and the community impact of suburbanisation) onto a sequence of chemical reactions and involving a subsequent mathematical analysis. Mapping the results back onto the use cases shows that one can retrieve a considerable amount of detail, making the modelling strategy general enough to be adaptable and scalable while also detailed enough to provide valuable insights for practical scenarios.

## Introduction

For decades now we know that understanding complex systems means a better grasping of who we are and our environment not just as individuals but as humanity^1^. Nowadays, in our increasingly interconnected world facing challenges like global warming, pandemics, economic and financial crises, the study of complex systems constitutes the foundation for understanding, modelling and predicting the dynamics of phenomena spanning on multiple scales and exhibiting emergent properties. This subject exploded in the last few years due in part to the availability and capacity to analyse Big Data^2^. However, in this study, we are mostly interested in highlighting the potential of an interdisciplinary approach to studying social complex systems and making their modelling and prediction process as accessible as possible.

While defining a complex system is not straightforward^3^ there are two main characteristics of complex systems in general: they are made of a large number of interacting entities or agents and they exhibit a non-intuitive collective behaviour^4^. Typically open, natural or social, complex systems are difficult to model because “one has to take into account at least two levels of abstraction (the micro and the macro levels) and the way they interact with each other”^5^. Practically, constructing an appropriate model of a complex system implies finding the right compromise between simplifying the system enough so it can be described by a series of laws and keeping enough details so that the model is still representative^4,6^.

In this paper, we propose using chemistry as the basis for the modelling method of complex systems. We use chemical reactions due to their characteristic ability to describe phenomena that occur on both macroscopic and microscopic scales hence providing two levels of abstraction for a given complex system. The goal of the modelling process is to derive and analyze the stability of the system^7,8^, in particular, the initial conditions and setups that lead to stable, oscillatory or chaotic dynamics^9,10^.

The study of complex systems is a well-established scientific field proposing numerous investigations into the realm of complex systems modelling, both from the epistemological^11^ and practical perspectives^12^. However, the majority of the existing research in the field is either purely analytical, lacking practical and ready-to-apply universal methods (e.g. some studies of financial system complexity emerged after the latest global financial crisis^13^) or employs stochastic modelling^14^. In this paper, we describe a deterministic method for assessing the stability of complex systems, marking the departure from conventional methods and offering a more flexible and user-friendly methodology.

The use of chemistry-based complex system models is not an entirely new research domain, especially in the field of economic modelling. There exist, for instance, chemical models of economic production^15^ or complex non-linear economic dynamics modelling using the chemistry toolbox^16^. However, the application and integration of these models have yet to gain widespread acceptance in the mainstream. At the same time, chemistry-inspired computing is gaining popularity in scientific workflow management systems because of its capacity to naturally express parallelism, distribution, and autonomic behaviours highlighted by some researchers^17^. The authors consider the chemical abstraction “as participating in the long-term objective of improving the workflow execution models on emerging platforms, like clouds, where the elasticity brings new modelling challenges” (ibid).

The novelty of this work consists in the fact that it aims to universalise (or democratise) the modelling process by ensuring that it can be constructed and used by individuals with a wide range of backgrounds and levels of expertise. This is achieved by building the method in such a way that is easy to apply to a diversity of complex systems addressing a wide variety of use cases. Moreover, the stability analysis is self-contained (as compact algorithms), it is computationally resource friendly to execute while the interpretation of the results is intuitive and impactful.

In order to illustrate the process of constructing the chemical model and conducting the mathematical analysis, we employ practical examples: the modelling of the interactions between customers and vendors in an economic market and to showcase the versatility of the method, we use the same model to derive valuable information in the field of project resource management. Additionally, we propose a cultural preservation use case to explore and underline even further complex behaviours such as feedback loops and uncertainty.

In all these cases, the purpose of the analysis is risk assessment and management: identifying the key factors playing a major role in the stability of the system, evaluating the uncertainties specific to a given scenario or decision and comparing the outcomes of narratives implying different initial conditions (e.g. economic and social landscapes, market conditions, business strategies).

The field of econophysics^18^ approaches economic systems from the perspective of e.g. statistical physics, complex systems, and network science, and addresses challenges such as extreme events, cascading effects or financial instabilities^19^. The proposed model employs one of the advantages of econophysics: it identifies and restricts the model focusing on the control variables needed to be representative of the use case while facilitating the analytical treatment of the economic system for predicting its stability^20,21^. Moreover, the chemical nature of the model provides the aiding step of describing the main interactions of the system and organising them in stages representative of the natural sequence of events, allowing for more realism. The cultural preservation case relates to urbanization, and econophysics modelling has already been applied to this field^22^ as well. In the current paper, we propose a less analytical and more practical modelling strategy that uses analogies with chemistry.

## Results

The premise of the **economic system** analysis centers on sustainability, more specifically on a producer’s challenge with waste management. We consider a producer engaged in the sale of agricultural goods (produce) like e.g. tomatoes.

This producer seeks to reduce or eliminate the waste which in this case are the imperfect tomatoes that fail to meet consumer preferences. The following bullet points represent the questions the producer is facing and the answers provided by the mapping method and the kinetic/stability analysis:

- Cost-effectiveness of waste reduction: Should the producer try to minimize waste by incentivising customers to purchase these imperfect goods? What are the potential financial implications of such a strategy? Specifically: can this method be implemented avoiding incurring losses? Our model shows that introducing incentivising strategies involving e.g tokens or vouchers, is an effective method to reduce waste. With a proper pricing strategy and a high demand for tomatoes, the initial economic system is not altered significantly.
- The financial viability of a hybrid approach: Is a combination of both strategies, involving incentivizing token-based purchases as well as traditional sales, economically viable? While the purely incentivised and not incentivised scenarios are economically stable in any circumstances, the hybrid system, for the cases that we have enough information about, does present a chance of instability. This case occurs when the demand for the product in general is restricted.
- Effective implementation of incentive mechanisms: How can the introduction of incentive mechanisms be implemented in a way that prevents the oversaturation of the market? Our predictions show that in the case when there is much more demand than supply, oversaturation of the market with vouchers/products or waste will not occur as long as the following conditions are met: 1. the quantity of favorable products is larger than the unfavorable ones and 2. the general value of the favourable products remains higher than the value of the unfavorable ones regardless of the incentivising method.
- Optimal pricing strategies: What are the pricing strategies for product categories that foster market stability and reduce waste? The optimal pricing strategy that insures stability in any scenario is the following: by buying waste products at a reduced price, customers earn tokens/vouchers that they can use to buy optimal products at a lower price than if they would not have the vouchers. At any point, the price of the waste products has to be lower than the price of the optimal products sold with vouchers. Moreover, the price of the optimal products sold with vouchers has to be lower than the price of the optimal product sold without vouchers.
- Economic assumption of the model: What key characteristics of the market play the decisive role in the outcome of this strategy? The economic assumptions behind the current market model include perfect competition, making both consumers and producers price-takers, meaning that neither of them has the market power to influence the prices. For the modelling process, it means that the prices are set as parameters at the beginning of the analysis and do not change as we examine the dynamics within the system. Another important assumption is that the consumers possess the necessary information on prices and the location of the goods in the market.

The context for the **team resource management** use case we are examining is a team working towards achieving certain milestones while having to incorporate new employees. With an increasing workload, the question is what is the maximum number of new team members that can be on-boarded with the least amount of disruption and what is the most effective strategy to support the newly forming team. To be more specific:

- Productivity optimisation: How many people can be on-boarded at the same time without causing significant disruptions to the team’s productivity and what is the main factor in insuring the smoothest integration of the new members? How many new team members can be incorporated in an existing team depends on the level of expertise the new members have to achieve and how this expertise is distributed among the existing team members. Generally our model shows that if the newcomers are more numerous than the existing team members, it is impossible to retain the efficiency of the team for tasks unrelated to the on-boarding process. For a successful and non-disputing integration, the new team members should be less than the possible mentors and ideally, they would have the basic knowledge and expertise covered by their previous experience so that the mentors have to spend minimal resources to finish joined/on-boarding tasks.
- Primary stability factors: What is the key factor that can lead to the instability of the system, in which conditions could this happen? The key factor that can make the difference between a stable and an unstable outcome is the restriction of resources (e.g. time, equipment, access to platforms etc.). According to our model, due to resource limitations, the processes most vulnerable (and prone to fail or not come to an end) are the ones requiring the most resources and the ones needing the longest time to be accomplished.
- Effective team support: How could an unstable scenario be prevented? What would effectively supporting the team mean? According to our mapping system the success of the entire resource management system (that insures that the processes are being completed) lies on three essential aspects: the most basic one is motivation of the team members that should be above a certain threshold, the efficiency of the team when presented with certain tasks (maximised by a good match between task difficulty and skill set) and the level of know-how present in the team. According to the nature of the tasks and functions performed by the team, any efficient support should maximise the three fundamental factors presented above.

The third example centers on the complex interactions between **urban sprawl and cultural preservation**. The members of a long-established rural community, thriving on cultural tourism for generations find themselves confronted with a challenging dilemma. They can either sell their properties at convenient prices to investors and urban developers ready to transform the village in the suburbia of a nearby metropolis or refuse and safeguard their community and traditions. As this is a profound decision for the entire collective, the main questions evolve around what the future holds:

- Villagers resist urbanization: Assessing the odds, Yes. Considering the size and the determination of the native community and well as their susceptibility in the face of the benefits offered by the urbanisation drivers, the villagers have the power to persuade the individuals considering selling their properties to change their minds.
- Outcome of the urban conflict: What is the key condition dictating whether the outcome of this situation is predictable or not? The equilibrium between the driving forces of the urban development process, including the resources and interests at play, and the scale and commitment of the community to stay true to its heritage will ultimately tip the scales in one direction or the other. Either the conflict resolves itself swiftly with the villagers as victors or the conflict will persist involving a long back-and-forth struggle.
- Large-scale strategy to mitigate urbanization: What the most effective strategy that can tilt the balance in the favour of the community? The most potent method to fight the urbanisation tendency is through an extensive outreach project that would rally as many interested people as possible in preserving the collective. The grater the size and the more dedicated the parties are to the cause, the higher the chances of prevailing in the conflict.
- Predicting possible outcomes: What is the realistic overview of all the potential scenarios? Given the influence of the urbanisation trend and the size and cohesion of the community, the outcome may lean toward either side. However, the decisive condition that determines the victory of one fraction over the other is challenging to quantify.

## Discussion

The presented use cases are general in nature. Even though the results obtained are intuitive, it is important to highlight that they do not include any data and are all derived from the modelling process itself, without any previous practical knowledge about the respective fields. Therefore the current findings should be taken as proof of concept rather than a quantitative study.

Due to the fact that complex systems are inherently non-deterministic, a deterministic method of investigation constitutes a major simplification of the modelled reality. The main emphasis of our study involves the finding of an optimal balance between a moderate level of simplification while still providing significant insights into the specific topics.

The current chemical model is not intended for a very detailed analysis of a complex system. We mean to underline its capacity to provide important information about a system and allow for comparing scenarios with different degrees of stability.

The mapping process is highly valuable as it inherently introduces a hierarchy among micro-level system elements. The macro-level mapping, which constitutes the primary process description, is specific enough to establish certain restrictions while remaining sufficiently flexible to accommodate the modelling of numerous and diverse types of interactions. How specific can the mapping and the model be in representing the complex system it is supposed to describe is ultimately up to the scope of the analysis and the system itself.

The current method can be efficiently used as an intermediate step in simulations like Agent Based Models (ABM) due to the compact form of the mathematical analysis and the fact that the initial mapping is necessary only once at the beginning of the simulation. Another application that we are currently exploring is to use the chemical model as a common sense proxy for artificial intelligence (AI) systems, particularly for extracting intuitive insights about the dynamics of complex systems that mirror human reasoning.

We are also currently investigating the possible applications of the method in circular economy on a global scale based on linked data principles (also called semantic technologies^23^). Our main focus is to harness the constructed knowledge graph to facilitate queries for matches involving companies with certain by-products and companies that could use these by-products as raw material^24^. The stability of the potential supply chain can be analysed using the chemical model. The advantage of using this method as an intermediary step in the matching process is that it not only provides general information about the possible success of the collaborations but can also explore alternative scenarios to optimise positive outcomes, dramatically increasing the impact and the outreach of the matching companies.

We are however aware of a list of possible drawbacks of the modelling method. We acknowledge the limited combination of coefficient values (1-3) and that interpretation of the coefficients’ time profile has to be adjusted to the use case.

Additionally, at this point, the mapping process is not yet automated and it can happen that it has to be done repeatedly to allow for certain complex systems. However, we are confident that large language models (LLM) will be able to aid in this process.

In conclusion, we found that the method itself is a robust tool for modelling and stability analysis of a wide range of complex systems at an optimal level of abstraction. It is tailored for a manageable number of processes per model but due to its scalability and versatility, we are convinced that the concept is ready to be implemented in simulations and used for real practical scenarios.

To draw a general limit of detail and analogy level for which the model breaks down is the scope of ongoing research.

## Methods

**Figure 1:**
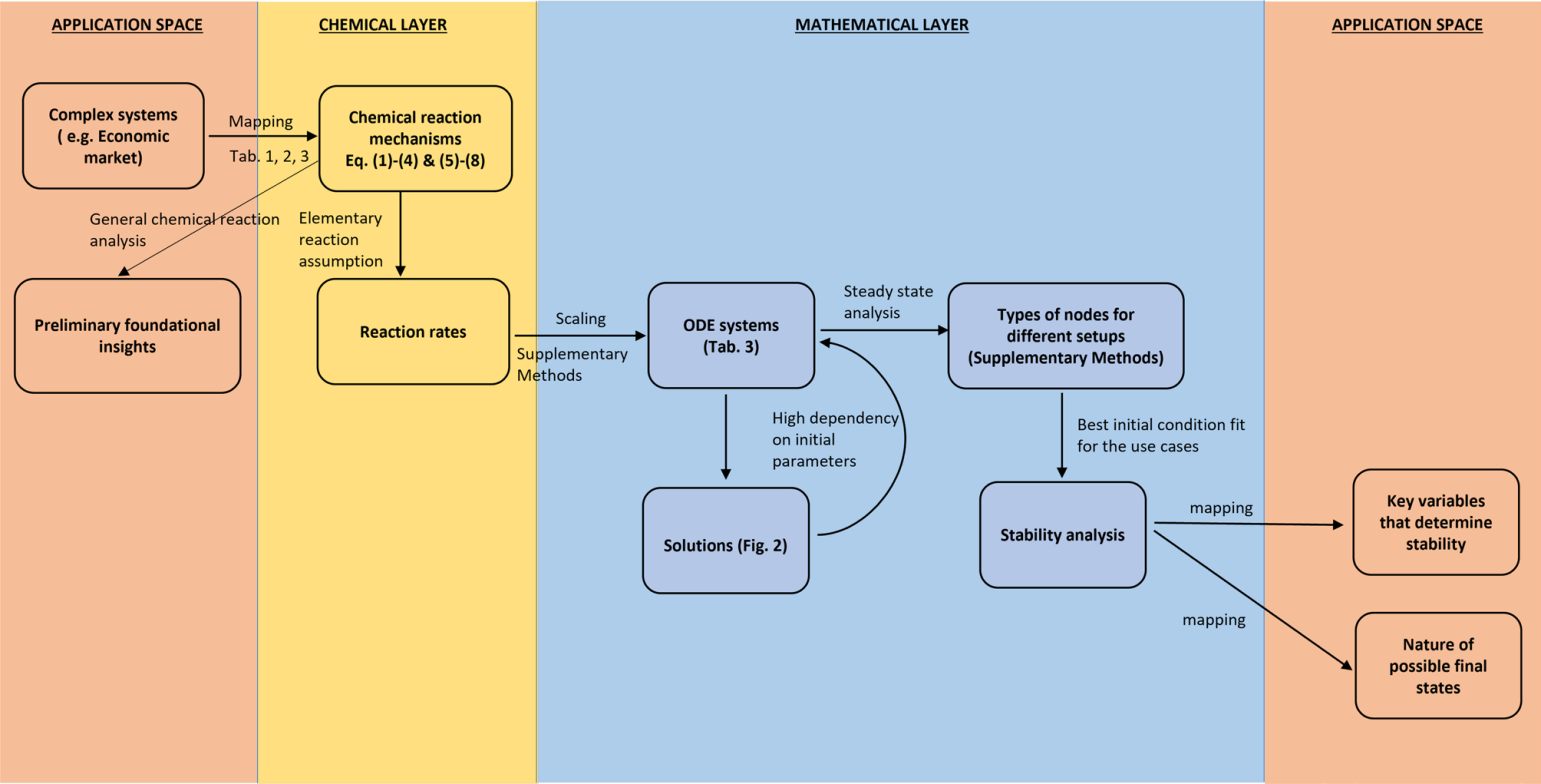
Overview of the conceptual layers the proposed method consists of. Every layer (marked by different colour areas) contains several steps (rectangles) while their logical sequence and connectivity are marked on the arrows. The interfaces between two conceptual layers are crossed either by mapping (equivalence of concepts) or scaling.

The chemical model that we propose in this paper contains several conceptual layers (see Fig. 1): the mapping of a complex system to a chemical reaction mechanism (of ideal gases); the characteristic reactions rates of each reaction are translated to an ordinary differential equation (ODE) system through scaling; the ODE system goes through a stability analysis and parameter study; the results are being mapped back to the original complex systems and the stability of the system is interpreted in the context of the original use case.

### Chemical layer

The chemical mapping of the initial complex system happens on two levels: a molecular level that insures that the physical quantities the reaction rates depend on have a clear and meaningful equivalent in the analysed application, and a macroscopic level at which the main interactions/processes are described in terms of chemical reactions. In order to use this method, one has to bring down the complex system to a series of basic processes setting the time frame at which they happen or at least their sequence.

For a better overview, Fig. 1 depicts the analysis steps and how they are intertwined, starting and ending with the practical application layer, the economic system and team resource management use case.

The first layer of the chemical model, the mapping on the chemical reaction mechanism of the first two use cases focuses on the interaction between the “product” ideal gas and the “money” ideal gas leading to the formation of “transactions”. Similarly, employees interact with resources to produce accomplished tasks. The basic reaction mechanism (referred to as CRM$_{1}$) can be written as:

1 $$\begin{aligned} Z \xrightarrow {{k}} \alpha X + \beta Y \end{aligned}$$

2 $$\begin{aligned} X + f A \xrightarrow {{k_{3}}} B + T \end{aligned}$$

3 $$\begin{aligned} Y + B + g A \xrightarrow {{k_{4}}} T \end{aligned}$$

4 $$\begin{aligned} Y + d A \xrightarrow {{k_{5}}} T \end{aligned}$$

The meaning of the notations representing chemical elements are listed in Table 1 while the coefficients are explained in Table 2.

**Table 1:** Overview of the chemical elements and their interpretations in the economic system and the team resource management system context.

Chem. Elem.	Economic market	Resource management
Z	The total mass of both types of goods, *X* and *Y*	The total team capacity
X	Imperfect good e.g. imperfectly shaped vegetables	New team member needing onboarding
Y	Sought-after product e.g. perfectly shaped tomatoes	Veteran team member
A	Unit of conventional currency	Unit of resources an employee needs
B	Token received by consumers purchasing a unit of	Task performed by a new employee needing
	Good *X* as a reward	Revision by the mentor
T	Successful financial transaction	Task accomplished successfully

**Table 2:** Overview of the coefficients of the reaction mechanism and their interpretations in the economic system and the team resource management system context.

Coefficient	Economic market	Resource management
$\alpha$	Coefficient corresponding to the part of the mass of the good *X* in the total mass	Total number of new additions to the team
$\beta$	Coefficient corresponding to the part of the mass of the good *Y* in the total mass	Total number of the initial team members
*f*, *g*, *d*	Correspond to prices of goods *X* and *Y* in different transactions	Set the amount of resources a type of employee needs for completing tasks

Deciding upon the values of the coefficients depends on the use case and the time profile of the first, second and third order reactions. E.g. for the economic system the larger the coefficient the lower the price while for the team resource management system the larger the coefficient the higher the amount of resources needed (see Supplementary Methods Fig. SI 1).

The reaction mechanism that embodies the optimal mapping of the third practical example, the cultural preservation issue, is the extensively studied Brusselator mechanism^25–27^, which will be referred to as CRM$_{2}$ (Br.):

5 $$\begin{aligned} A \xrightarrow {{k_{6}}} X \end{aligned}$$

6 $$\begin{aligned} B + X \xrightarrow {{k_{7}}} Y + D \end{aligned}$$

7 $$\begin{aligned} 2X + Y \xrightarrow {{k_{8}}} 3X \end{aligned}$$

8 $$\begin{aligned} X \xrightarrow {{k_{9}}} E \end{aligned}$$

The meaning of the notations representing chemical elements of the Brusselator mechanism are listed and explained in Table 3.

**Table 3:** Overview of the chemical elements and their interpretations in the cultural preservation context.

Chem. Elem.	Cultural preservation
X	Local villagers adamant on staying and preserving the culture and traditions of the community
Y	Villagers entertaining the idea of of selling their properties and relocating
A	Village community
B	Companies interested in the urban development project making the financial offer for relocation
D	By-standards effected by the relocation process
E	Villagers who leave the community for other reasons than urban development

Practically, the second layer of the chemical mapping consists in finding the equivalent concepts between the reaction rate equation and the applications. The reaction rate equation can be written as $r_{rate} = \rho Z_{AB} f$, where $\rho$ is the steric factor, accounting for the orientation of the collisions, $Z_{AB}$ is the collision density and a function of the reactant concentrations and *f* is the fraction of molecules with enough energy to overcome the activation barrier (a function of the activation energy of the reaction $E_{A}$). In the application layers the activation energy represents the willingness and the possibility of the customers to purchasing the goods, the motivation of the team in solving tasks and the interest in either keeping the community together or drive the urban development plan. Building on this basis, the collision frequency’s equivalent is the actual density of the customers on the market, the efficiency of the employees, the scale and the determination, for various reasons, of the two fractions clashing in the conflict between urbanisation and local resistance. Once the atoms/molecules are above the activation energy and the collision frequency is adequate for the reactions to take place, the last restriction is the angle under which the collisions happen: the information available to the customers on the market, the specific know-how of the field for the team members and on one hand how deeply the community is rooted in traditions and culture and on the other hand the relative significance of the compensation for relocation in the context of the local generational and situational poverty.

We consider for this set of use cases that the reactions are elementary. However, non-elementary reactions can also be used for the modelling process^28^.

Another important feature of the model is that there is not enough information about which reaction is the rate determining step (the reaction that is the slowest and determining the rate of the entire reaction mechanism), and hence the rate equations will be solved simultaneously, leading to the reaction rates describes in Table 4. In the same table we depict the equivalent scaled ODE systems making the connection between the Chemical and the Mathematical layers through a scaling step. The approximations regarding the values of $k_{1} =... = k_{9} = 1$ $(\frac{mol}{Ls})^{-1}$ as well as the scaling applied to obtain the ODE systems are described in Supplementary Methods online.

**Table 4:** The reaction rates of the reaction mechanisms CRM$_{1}$ and CRM$_{2}$ (Br.) as a non-linear first and second order ODE systems and their scaled versions.

Reac. Mec.	Reaction Rate ODE sys.	Scaled ODE sys.
CRM$_{1}$	$\frac{d[X]}{dt} = k[Z]^{1/\alpha } - k_{3}[X][A]^{f}$	$x' = z^{1/\alpha } -xa^{f}$
	$\frac{d[Y]}{dt} = k[Z]^{1/\beta } - k_{4}[Y][B][A]^{g} - k_{5}[Y][A]^{d}$	$y' = z^{1/\beta } -yba^{g} - ya^{d}$
	$\frac{d[A]}{dt} = -k_{3}[X][A]^{f} - k_{4}[Y][B][A]^{g} - k_{5}[Y][A]^{d}$	$a' = -xa^{f} -yba^{g} - ya^{d}$
	$\frac{d[B]}{dt} = k_{3}[X][A]^{f} - k_{4}[Y][B][A]^{g}$	$b' = xa^{f} -yba^{g}$
CRM$_{2}$ (Br.)	$\frac{d[X]}{dt} = k_{6}[A] - k_{7}[B][X] + k_{8}[X]^{2}[Y] - k_{9}[X]$	$x' = a - bx + x^{2}y - x$
	$\frac{d[Y]}{dt} = k_{7}[B][X] - k_{8}[X]^{2}[Y]$	$y' = bx - x^{2}y$
	$\frac{d[A]}{dt} = - k_{6}[A]$	$a' = -a$
	$\frac{d[B]}{dt} = - k_{7}[B][X]$	$b' = -bx$

### Mathematical layer

The process of solving the ODE system, constituting a major part of the mathematical layer, takes different forms and routs for different models. In the current example, the solutions of the CRM$_{1}$ ODE system are not stable, hence the need for a steady state analysis and more simplifications (see Fig. 1). However, it is our opinion that the steady state analysis offers so much insight in itself that it should be done whether the ODE system has stable solutions or not.

The complete symbolic analysis of the steady state solutions and the nature of the critical points according to the constants (elements found in abundance) and variables can be found in Supplementary Methods Table SI 1. Taking into account the conditions for certain types of solutions, we can determine the most relevant systems for the specific use cases presented.

For CRM$_{1}$, mapping the economic market and the team resource management use cases, we assume that *A* is fixed (considering it is found abundantly, at levels orders of magnitude higher than the other elements and in alignment with the perfect competition assumption) while *X*, *Y* and *B* are variable. Carrying out the steady state analysis, in order to insure that the solution is positive it was found that the condition $\alpha > \beta$ has to be fulfilled and thus the steady state solution is of a saddle node type. This suggests the presence of complex dynamics with both stable and unstable behaviour, depending on which side it is approached from. Changes along an axis will lead the system towards equilibrium, while from another direction, changes will drive the chemical system further from equilibrium.

**Figure 2:**
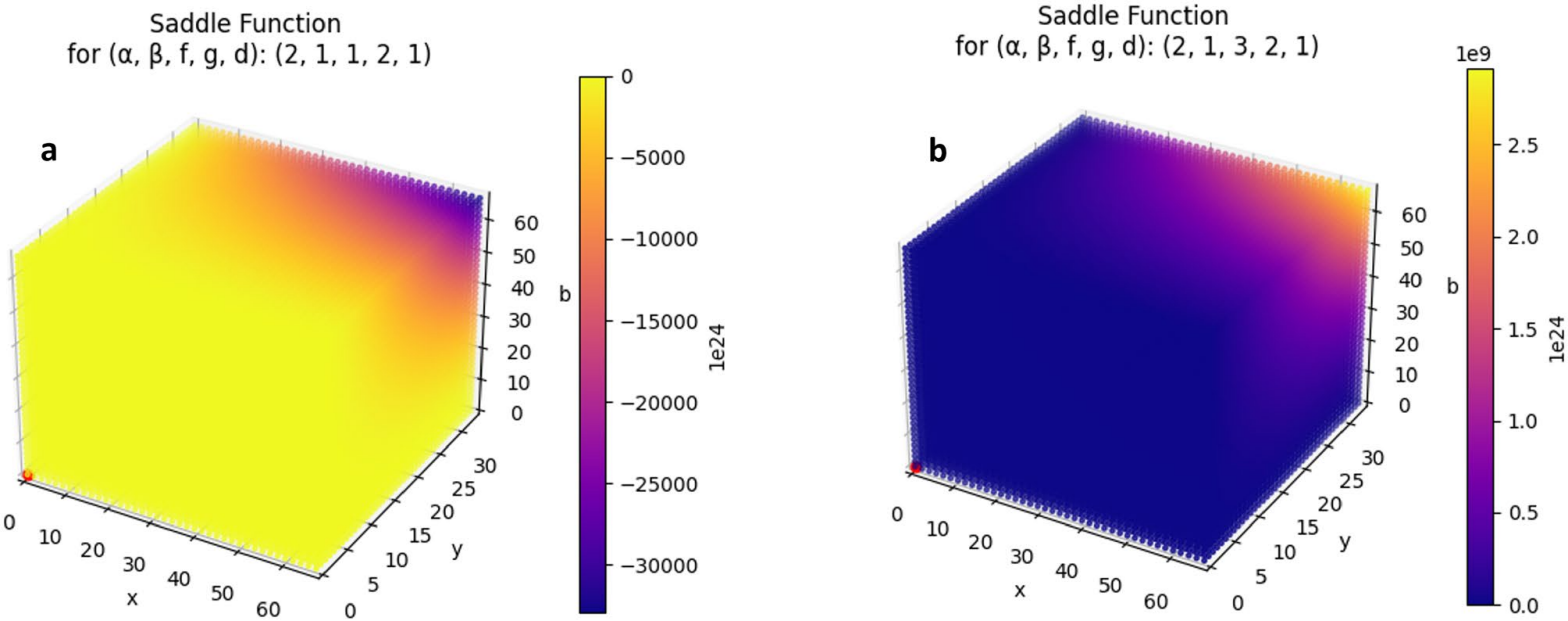
Saddle point functions with the following fixed parameters: $z = 100$ and $a = 10000$. In the left plot the steady state solution (red dot) is a local maximum and in the right plot the steady state solution (also red dot) is a local minimum.

A closer look reveals two types of saddle functions (describing the vicinity of the saddle-node) due to the high sensitivity of the steady state solution to the initial value (concentration) of *A*. However, when focusing only on the positive quadrant of the function around the steady state solution, meaning that all the quantities are positive at all times, we obtain two different regimes: a state of stable and one of unstable equilibrium (see Fig. 2). Stable equilibrium (Fig. 2b) is obtained through the following coefficient combinations: ($\alpha$, $\beta$, f, g, d) = (2,1,3,2,1), (3,2,3,2,1), (3,1,3,2,1).

A crucial observation about the functions around the stable equilibrium nodes in the positive quadrant is that the amount of *A* in the system seems to strongly influence the shape of the functions. The restriction of the amount of *A* initially present at the beginning of the chemical reactions causes a transition behaviour in the function itself. Of course the restriction of the initial amount of *A* will effect vicinity of the unstable equilibrium nodes as well but it will not change their general shape.

The fact that *A* is not a variable of the saddle point function is a condition of this steady state analysis, however, it seems like the vicinity of stable equilibrium nodes are very sensitive to the initial value of *A* determining the system to go through a transition phase (with a local minimum and a local maximum close to each other) and by restricting *A* even more, the steady state solution changes its characteristics from stable to unstable (see Supplementary Methods Fig. SI 2).

**Figure 3:**
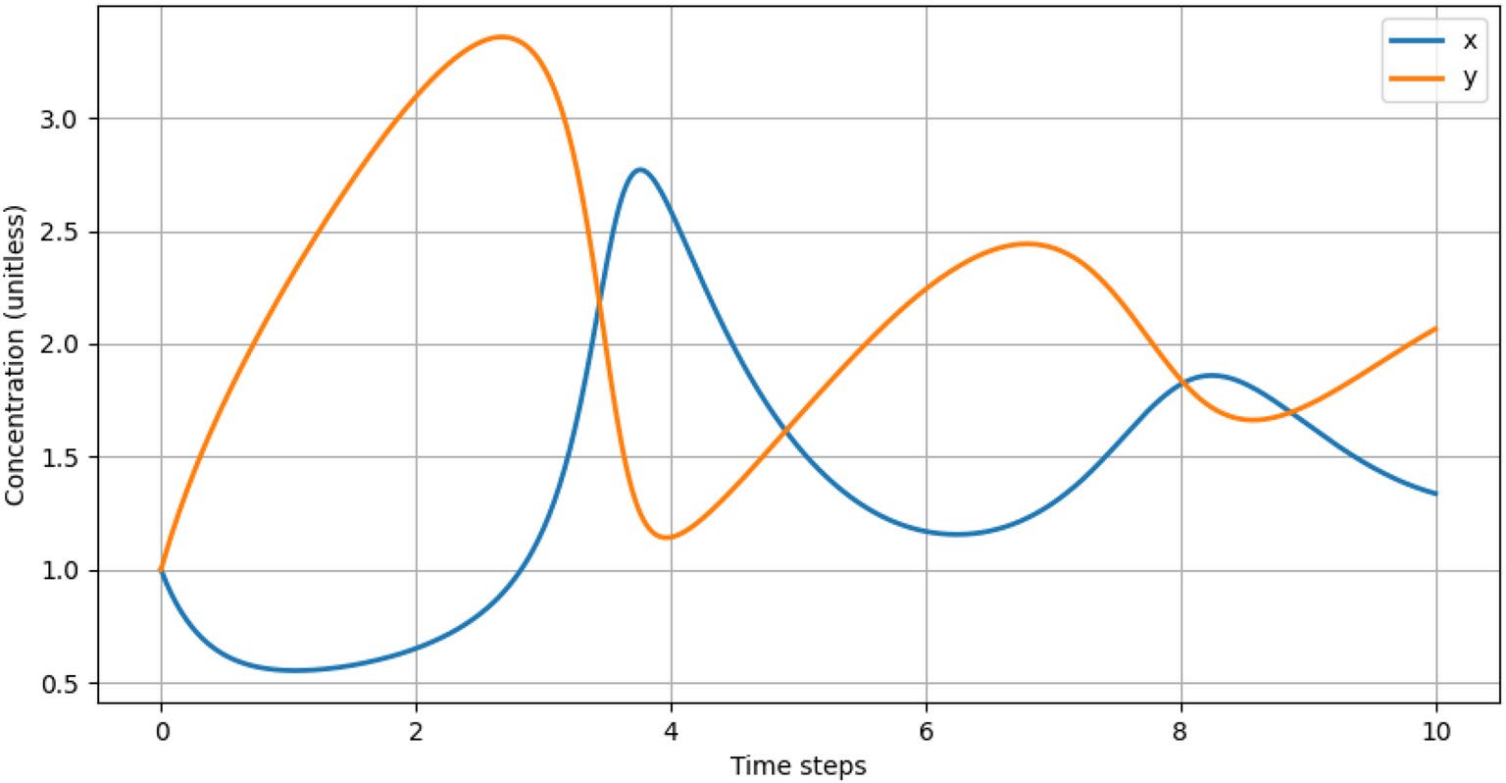
Brusselator oscillations obtained by the following initial conditions: $a = 1.5, b = 3, x(0) = y(0) =1$. The pattern and emergence of the feedback loops may be interpreted as self-organisations.

For the CRM$_{2}$ (Br.) system, depicting the cultural preservation dilemma in the face of suburbanisation, we consider that *A* and *B* are present in abundance at the beginning of the reactions and focus on *X* and *Y* as variables. At equilibrium, the solution $S(x_{sol}, y_{sol}) = (a, b/a)$ and the oscillatory behaviour of *x* and *y* over time is depicted in Figure 3.

Carrying out the steady state analysis of the Brusselator system we learn that the condition $b = a + 1$ constitutes the threshold two qualitatively distinct behaviours. In other words, at this point ($b = a + 1$) the system exhibits a transition between stability and instability (or a split) called Hopf bifurcation^7,29,30^.

### Application layer

In this subsection we will shine light on the thought process behind the interpretation of the chemical reactions and the subsequent mathematical analysis which leads to the statements presented in the Results section.

The initial step in the interpretation process involves examining the entirety of the chemical reaction mechanisms. This tactic, implemented prior to starting the mathematical analysis, can provide significant preliminary foundational insights.

By scrutinizing the $CRM_{1}$ mechanism, focusing on the net reaction:

9 $$\begin{aligned} Z + [\alpha (f+g-d)+\beta d]A \xrightarrow {\vphantom{0}} (\alpha + \beta )T \end{aligned}$$

one can attempt to assess, in a general way, the cost-effectiveness of the waste reduction strategy. It becomes evident that the net reaction does not yield any product type (perfect or imperfect), indicating that at the end of the reactions, or of a market day, there is no residual waste. Hence, the strategy of incentivising the sale of imperfect goods in this manner is an effective one. Moreover, when comparing the same net reaction (Eq. 9) with the net reaction of the mechanism that does not contain incentives (described in^28^):

10 $$\begin{aligned} Z + [\alpha c+\beta d]A \xrightarrow {\vphantom{0}} (\alpha + \beta )T \end{aligned}$$

it can be noted that the two reactions become equivalent when $c = f + g - d$. As the coefficients involved in this use case correspond to the prices of the goods (see Table 2), we can conclude that a fitting pricing strategy can insure that the amount of money involved in the reaction mechanism (*A*), or in the total transactions of a market day, remains unaffected by the introduction of incentives.

This comparison touches upon the effective implementation of the incentive mechanism as well: as long as the vouchers or tokens (or product types e.g. *X*, *Y*) are absent from the net reaction, there is no peril of oversaturation, at least on the short run.

Because the team resource management example shares the mapping onto the same $CRM_{1}$ mechanism, we can also show that a careful management of resources can insure that there should be no significant changes in overall productivity when all the reactions rates involved are comparable.

The general structure of the $CRM_{2}$ (Br.), especially the autocatalytic equation (6), outlines a nonlinear feedback mechanism due to the coefficient 2 in front of *X* (complemented by the elementary reaction supposition). This nonlinearity leads to the emergence of oscillatory behaviour and potentially chaos, both being hallmarks of systems complexity. The situation that was mapped onto this equation illustrates how two villagers can influence another villager’s decision to leave the collective, potentially convincing them to stay.

The feedback loop itself is even better illustrated in Fig. 3 where the solution of the simplified $CRM_{2}$ (Br.) system is shown as a function of time. The fact that the amount of *x* and *y* is raising and falling periodically depicts the potential for the self-organisation of the system as a whole. In reality, observers of the conflict will witness the number of villagers advocating for cultural preservation swell as the opposition to selling and leaving increases. Conversely, the pro-cultural preservation coalition may decline as more individuals are drawn to the side that considers relocation.

Solving both ODE systems (see Table 4) along with the assessment of their characteristics and the steady state analysis provide a remarkable amount of information. The stability analysis of $CRM_{1}$, for the carefully chosen variable configuration, reveals that in order to obtain a positive solution the condition that $\alpha > \beta$ has to be fulfilled. This condition, in chemical terms means that the rate of production of *Y* has to be higher than the production rate of *X*. In other words, the rate at which *Y* becomes available to be sold should be higher than the rate of *X*. Practically this condition insures that the less favourable products *X* are present in a lower quantity than the perfect products *Y* and consequently that the new team members should be less than the veterans for successful integration.

The stability analysis conveys another important finding: certain pricing strategies result inherently in stable equilibrium when *A* is unlimited and namely $f> g > d$. Due to the fact that the coefficients can take only values from 1 to 3, this condition restricts $f=3, d=2, g=1$. This means that the good *X* is the cheapest one, *Y* has a higher price when tokens are part of the transaction, and that the good *Y* is the most expensive when bought without incentives. This outcome serves as validation because it is indeed the most economically sound pricing choice.

Mathematically speaking, the notion of stability is linked to the behaviour of solutions over time, more precisely to how small disturbances (e.g. perturbations in initial conditions) lead to significant changes in the studied solution. In chemistry, the notion of stability is used to describe the state of a system at its lowest energy point, where it tends to resist concentration fluctuations over time.

The stability of an equilibrium point (also known as a node, critical point or steady-state solution) refers to the tendency of small perturbations in the vicinity of the steady-state solution to converge or diverge from the equilibrium point. Even though it might seem like the notion of stability is quite abstract, we can interpret it in terms of the use cases we are examining.

The fact that the steady state solution is so sensitive to the value of *A* and that the restriction of *A* can induce instability in an otherwise stable situation (see also Supplementary Methods Fig. SI 2) means that the demand restriction, more precisely the constraint of the amount of money present at the beginning of the market day, is a key factor that determines the stability of the system. The same way the limitation of resources that employees need for carrying out their tasks is the primary element in deciding the stability of the integration strategy.

The economic interpretation of the concept of stability refers the outcome after a day when the transactions naturally stop happening. The outcome will be considered stable in case there is no excess demand/supply, so there are no consumers left on the market searching for goods and no producers stranded with unsold goods. Consequently instability will be treated as a volatile state of the market in the sense that small changes can lead to unpredictable leftovers or over-saturation. In the case of resource management stability will be used as predictable outcome of the implemented strategy with all the resources consumed and all the planed tasks accomplished.

Similarly, the stability analysis of the $CRM_{2}$ (Br.) system reveals that the bifurcation threshold is given by the condition $b = 1 + a^{2}$ or the point where the scale of suburbanisation is almost balanced out by the scale the community, squared. The (Hopf) bifurcation is the critical point that marks the transition between a stable and a oscillatory (and even chaotic) behaviour. In the urbanisation and cultural preservation paradigm these outcomes reflect a state with a clear outcome (the villagers or the development companies won) for a stable solution, a state of continuous conflict between the two fractions - the oscillatory behaviour or a completely unpredictable end brought about by an unexpected factor - the chaotic behavior.

How much stronger the influence of urbanisation is in comparison to the community size and cohesion can determine the way a solution is approached. The relevant difference between a node and a focus point (stable or unstable) for the given use case is that the trajectory towards a node is directed (or radial) towards the solution (stable node) or away from the solution (unstable node) while the trajectory towards a focus point is spiraling onwards (stable focus) or outwards (unstable focus). This distinction is essential as it shows the process we can expect leading to a resolution and whether factors like politics or media coverage can make a difference or not in the final outcome.

Last but not least, the micro level mapping can also offer a significant amount of valuable information. Due to the fact that the reaction rates determine how fast and how effective certain chemical equations are, the interpretations of what the reaction rate components are in addition to the already available information from the net reactions and the steady state analysis, can determine the key elements for the complex systems we are analysing.

In the economic market case, the molecular level mapping and the initial assumptions impose perfect competition and a high enough willingness and possibility for the customers to purchase goods on the market. The more numerous the customers the better and the better they are informed about products the more efficiently the market will flourish. The outcome of the team resource management strategy success depends on the team’s efficiency in tasks solving, which in turn depends on whether the challenges align with their skills. Additionally, the team’s collective expertise is a decisive factor in their success.

In the case of the urban sprawl and cultural preservation dilemma we can take this mapping one step further and not only name the key factors that determine the flow of the system but also deduce some strategies that might benefit one side or the other. The elements that can influence the outcome of the conflict are scale and the commitment of the two sides to their cause, the profound connection of the community to its traditions and culture and the importance of relocation compensation against the current social and financial situation of the collective.

One possible strategy in raising the amount of *X* and hence the traditional villager community in the detriment of the people settled on relocation is to either increase the rate of equation (5) and/or equation (7). Practically this could mean finding outside investors that are committed to the case of keeping the collective together just as much as the original villagers. One could think of a large scale media outreach for the cause in particular or for certain traditional local “treasures” (e.g. a traditional dish or custom, an endemic plant or animal species etc.) that would otherwise disappear.

## Supplementary Information

Supplementary Information.

## Data Availability

The datasets generated and/or analysed during the current study are available in the Democratising-Complex-System-Modelling repository, https://github.com/pardianabele/Democratising-Complex-System-Modelling.

## References

[1] Meadows, D. H. E. *The Limits to Growth : A Report for the Club of Rome’s Project on the Predicament of Mankind* (Universe Books, New York, 1972).

[2] San Miguel, M. Frontiers in complex systems. *Front. Complex Syst.*10.3389/fcpxs.2023.1080801 (2023).

[3] Estrada, E. What is a complex system, after all?. *Found. Sci.*10.1007/s10699-023-09917-w (2023).

[4] Boccara, N. *Modeling Complex Systems* (Springer, New York, 2010).

[5] Siebert, J. What are complex systems? - understanding and assessing complex phenomena. *FRAUNHOFER IESE* https://www.iese.fraunhofer.de/blog/complex-systems/ (2022).

[6] Vemuri, V. *Modeling of complex systems: An introduction* (Academic Press, Liverpool UK, 1978).

[7] Epstein, I. R. & Pojman, J. *An Introduction to Nonlinear Chemical Dynamics Oscillations, Waves, Patterns and Chaos* (Oxford University Press, New York, 1998).

[8] Strogatz, S. H. *Nonlinear Dynamics and Chaos* (Perseus Books, Reading, 1994).

[9] Field, R. J. Chaos in the Belousov–Zhabotinsky reaction. *Mod. Phys. Lett. B.*10.1142/S021798491530015X (2015).

[10] Kol’tsov, N. Chaotic oscillations in four-step chemical reaction. *Russ. J. Phys. Chem. B* **11**, 1047–1048. 10.1134/S1990793117060045 (2017).

[11] Edgar, M. *On complexity* (Hampton Press, Cresskill, 2008).

[12] Aumann, C. A. A methodology for developing simulation models of complex systems. *Ecol. Model.* **202**, 385–396. 10.1016/j.ecolmodel.2006.11.005 (2007).

[13] Dekker, S. W. Drifting into failure: Complexity theory and the management of risk. In *Chaos and complexity theory for management: Nonlinear dynamics*, 241–253 (IGI Global, 2013). 10.4018/978-1-4666-2509-9

[14] Castellani, B. & Rajaram, R. Past the power law: Complex systems and the limiting law of restricted diversity. *Complexity* **21**, 99–112. 10.1002/cplx.21786 (2016).

[15] Padgett, J. F. E. A. Economic production as chemistry. *Ind. Corp. Change* **12**, 843–877 (2003).

[16] Burina, E. Chemistry at the service of economic modelling: Vladimir Bazarov’s approach to formalizing business cycles. *Oeconomia* **13**, 995–1027 (2023).

[17] Fernandez, H., Tedeschi, C. & Priol, T. A chemistry-inspired workflow management system for scientific applications in clouds. In *2011 IEEE Seventh International Conference on eScience*, 39–46 (IEEE, 2011).

[18] Stanley, M. H. R. E. A. Scaling behavior in the growth of companies. *Nature* **379**, 1476–4687. 10.1038/379804a0 (1996).

[19] Rosario, M. N. & Kertész, J. Focus on statistical physics modeling in economics and finance. *New J. Phys.* **13**, 025011 (2011).

[20] Helbing, D. *Social Self-Organization, Agent-Based Simulations and Experiments to Study Emergent Social Behaviour* (Springer, Heidelberg, 2012).

[21] Korecki, M. E. A. Democratizing traffic control in smart cities. *SSRN*10.2139/ssrn.4598461 (2023).

[22] Rosser Jr, J. B. Econophysics and entropy in dynamically complex urban/regional systems. In *Foundations and Applications of Complexity Economics*, 89–100 (Springer, 2021).

[23] Fürber, C. *Semantic Technologies* (Springer Fachmedien Wiesbaden, Wiesbaden, 2016).

[24] Bayerisches Staatsministerium für Wohnen, B. U. V. Umweltschutz im winterdienst mit salzwasser aus der gurkenproduktion. *online at* https://www.stmb.bayern.de/assets/stmi/vum/strasse/betriebsundwinterdienst/41_flyer_gurkenwasser.pdf/ (2021).

[25] Prigogine, I. *From Being to Becoming: Time and Complexity in the Physical Sciences* (W. H. Freeman, 1980).

[26] Ruth, M. & Hannon, B. *The Brusselator* (Springer, New York, 1997).

[27] Alshammari, S., Al-Sawalha, M. M. & Humaidi, J. R. Fractional view study of the Brusselator reaction-diffusion model occurring in chemical reactions. *Fractal Fract.*10.3390/fractalfract7020108 (2023).

[28] Pardi, A. & Paolucci, M. A chemical analysis of hybrid economic systems-tokens and money. *Mathematics*10.3390/math9202607 (2021).

[29] Rovinsky, A. & Menzinger, M. Interaction of turing and hopf bifurcations in chemical systems. *Phys. Rev. A* **46**, 6315–6322. 10.1103/PhysRevA.46.6315 (1992). 9907943 10.1103/physreva.46.6315

[30] Yan, X.-P., Zhang, P. & Zhang, C.-H. Turing instability and spatially homogeneous hopf bifurcation in a diffusive brusselator system. Nonlinear Anal. Model. Control. 10.15388/namc.2020.25.17437 (2020)

