# Supplementary material for: Democratising complex system modelling
Source: Sci Rep. 2024 May 8;14:10590. doi: 10.1038/s41598-024-61318-6 (PMC11634906; doi:10.1038/s41598-024-61318-6)
Supplement: Supplementary file 1 — Supplementary Information. [file 41598_2024_61318_MOESM1_ESM.pdf]

Supplementary Methods

Coefficient profiles for CRM_1

The reactions of all the chemical reaction mechanisms are assumed to be elementary, meaning that the chemical species react directly to form products in a single reaction step. This also means that the coefficients of the chemical species will have a direct influence on their reaction rates (mathematically speaking the coefficients will become powers of the chemical species when writing the reaction rates).

For elementary reaction (in the CRM_1, economic market scenario), the concentration variation in time of e.g A can be written as $ln[A] = ln[A]_0 - k \cdot t$ for first order reactions, $1/[A] = 1/[A]_0 + kt$ for second order reactions and $[A] = [A]_0/\sqrt{(}2 \cdot k \cdot t[A]_0^2 + 1)$ for third order reactions. In our model we consider that the coefficient of A is setting the price of the goods while A is being a unit of currency. However, the equivalence between the coefficients of element A and the price of a good is not straight forward.

In the calculation of the concentration variation of A in time (see Fig. SI 1), k is considered to be constant and 0.5 s^{-1} and $[A]_0$ is assumed to be $10(\times 10^{-20} mol/L)$.The concentrations in the current paper will have the same type of unit due to the fact that we approximate that a medium sized business would handle in one market day about $6,022 \times 10^4$ units of product or currency (equivalent here to number of particles).

Firstly, as depicted in Figure SI 1 showing how the money "gas" would react with the goods to form transaction, one can see how the higher the coefficient of A, the steeper the concentration of A decreases in time. From en economic point of view, the steepest profile of A (as a third order reaction) should corresponds to the cheapest good. Similarly, the profile that has the mildest slope (the first order reaction) corresponds to the most expensive good.

Thus, lower coefficients result in slower reactions, and since the coefficients relate to prices, the mapping should be explained in more detail here. Lower coefficient means higher price, not the other way around. This interpretation had to be adopted in order to conserve the logic and coherence of both systems, chemical and economic.

Secondly, we only consider the values 1, 2 and 3 for the coefficients of the reaction mechanism because higher values than 3 are very unusual in reality. More than 3 atoms colliding at the same time to take part at a reaction is a very unlikely event. From the economic point of view, the three different values are enough to model three different prices corresponding to CRM_1. A coefficient of 5, for example, would correspond to extremely low price, making a good almost free compared to others. Stoichiometry forces us to deal only with integers, which restricts the set of possible relative prices analyzed. However, it provides a good starting base for the further research development and results in quite a wide range of cases to study.

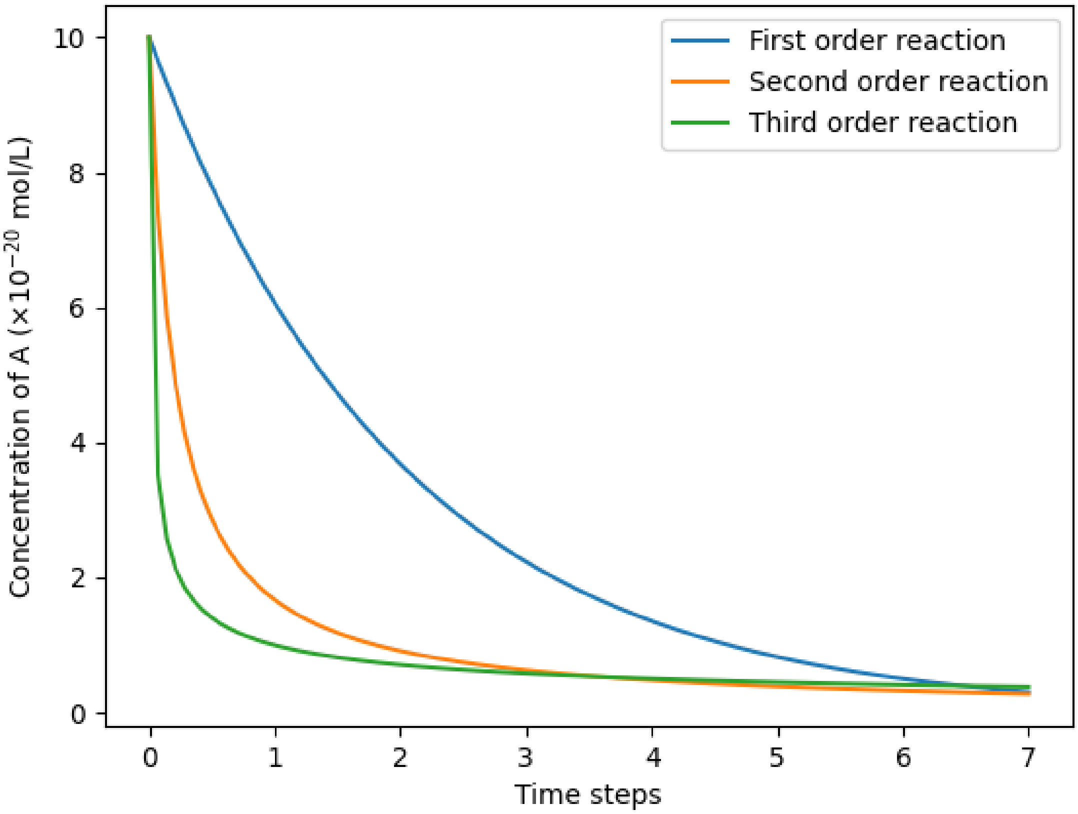

Figure SI 1: The time dependent concentrations of A when A's coefficient is 1 (blue line, first order reaction), when A's coefficient is 2 (orange line, second order reaction) and when A's coefficient is 3 (green line, third order reaction). The higher the order of the function, the steeper the concentration decreases in time.

On the other hand in the case of the team resource management example, the coefficients determine the amount of resources needed by an employee and hence, the equivalence of low coefficient - low amount of resources is sufficient.

Assumptions and scaling of the ODEs

One of the most important assumption of the model is that there is no information about which reaction is the rate determining step (the reaction that is the slowest and that determines the rate of the entire reaction mechanism), and hence the rate equations will be solved simultaneously. From the economic point of view this means that in the beginning of our analysis we do not know which transaction is going to happen faster. In other words, for CRM_1, there is no predetermined customer preference for any of the goods X or Y. This assumption is valid when writing down the reaction rates describes. Every reaction of CRM_1 and CRM_2 (Br.) has a reaction rate coefficient (k) but in our particular case we do not have

any information about these coefficients.

However, there are two arguments for our current assumptions regarding k: 1) the assumption that all the reactions happen almost simultaneously meaning that the reaction rate coefficients, even though not equal to each other, are similar enough to be treated as equal ($k_1 = ... = k_9 = k$), or 2) that the different reaction rate coefficients are incorporated into the scaling method that allows for writing the unitless time and concentration variables x, y, z, a, and b and the scaled ODE system in the right column of Table 4. The scaling steps and the factors are described in [1] Appendix C.

By having two reactions with Y as reactant, eq.(3)-(4), and not knowing which one is slower than the other (or any information about a preference for one or the other reaction), we will balance stoechiometrically each individual reaction but not the reaction mechanism as a whole. This way α and β coefficients set a relative initial concentration for X and Y but do not force any certain fraction of Y to take part at eq.(3) or eq.(4).

Symbolic analysis of the steady state solutions

The steady-state analysis of an ODE system is generally carried out to determine the long-term behaviour of the system, even if a solution to the system already exists. This can be very useful in determining the overall stability and performance of the system.

The dependence of a system on certain initial conditions is known as sensitivity to initial conditions. Systems that are sensitive this way are said to have a large degree of chaos. By doing the steady-state analysis, one can understand how the system behaves over time and identify potential areas of concern or instability by studying the sensitivity to initial conditions.

In [1], a symbolic study was done about the type of solutions and critical points of the CRM_1 ODE system. This means that the CRM_1 ODE system was analysed while keeping all coefficients as general notations without giving them specific values. Table SI 1 lists not only the types of solution and node of the generic CRM_1 ODE system (No. 1) but also all the simplified cases in which one or several of the initial variables (x, y, a, and b) are fixed. These simplified ODE systems are interesting because they can still capture scenarios relevant in an economic and team resource management context. On the other hand, present only one CRM_2 (Br.) case due to the very good fit of the cultural preservation issue case and because this particular scenario was extensively studied in the literature.

As found in the [1] analysis (Table A3 from Appendix D), only some of the steady states can be found symbolically. Cases 1, 2, 4, 5, 7, 9 and 11 from Table SI 1 have two rational, positive solutions but only for specific coefficient values and certain initial conditions.

Transition phase and sensitivity to A

The fact that A is not a variable of the saddle point function is a condition of this steady-state analysis. However, the stable functions are very sensitive to the initial value of A. Figure SI 2 shows a series of functions (on the left $A = 10 \times Z$, in the middle $A = Z$ and on the right $A = Z/10$). It seems like when restricting A, the system goes through a transition

phase (with a local minimum and a local maximum close to each other), and by restricting A even more, the stable function gains the specific characteristics of an unstable functions.

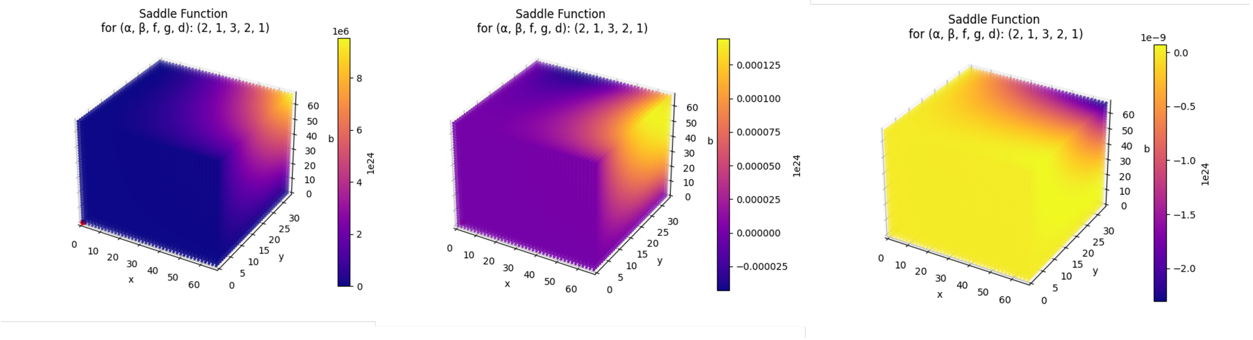

Figure SI 2: Stable solution case of $(\alpha, \beta, f, g, d) = (2, 1, 3, 2, 1)$ with $A = 10 \times Z$ (left), $A = Z/2$ (middle) and $A = Z/10$ (right). Lowering A leads to the function to acquire unstable characteristics.

The transition phase shown in Figure SI 2 middle looks like a saddle-node bifurcation but at this point in the analysis, there is a lot more effort needed to be able to describe this transition accurately. This particular situation can be an indication of chaotic behaviour or just high sensitivity of the steady state solution to A. However, for our market use case, finding the threshold value of A for which major changes in the behaviour of the steady state solution start to happen is more important than finding what exactly determines this behaviour (or where would even stronger restrictions of A lead).

This finding is important because, as it turns out, the system is only stable if sufficiently many consumers come to the market with conventional currency at the beginning of the market day. This conclusion is quite intuitive since the steady state solution (when everything is sold) will be more stable if there are always consumers to buy extra units, even added at the end of the market day. However, the model helps explore this further and determine where the turning point from stable to unstable might happen.

The hyper-sensitivity of the steady state solution to a fixed parameter can be a sign of chaotic behaviour or of some bifurcation, but as stated above, exploring in more detail this behaviour does not bring much more practical information about the market dynamics, especially because we assume that there is more than enough money in the system.

[1] Pardi, A. and Paolucci, M., ”A Chemical Analysis of Hybrid Economic Systems—Tokens and Money” 2021, Mathematics, 9, https://www.mdpi.com/2227-7390/9/20/2607, 10.3390/math9202607

Reac. Mec.	No.	Fix.	Var.	Sol.	Cnd.	Tr(J), Det(J) S(J)	Cnd.	Node type
	1	-	X, Y, A, B	(+,+,+) exp.	-	-, 0 , +	-	degenerate
	2	B	X, Y, A	(+,+,+) exp.	-	-, 0 , +	-	degenerate
	3	A	X, Y, B	(+,+,+)	[1]	-, - , +	-	saddle
	4	Y	X, A, B	(+,+) exp.	-	-, - , +	-	saddle
CRM_1	5	X	Y, A, B	(+,+) exp.	-	-, - , +	-	saddle
	6	A, B	X, Y	(+,+)	[2]	-, + , +	-	stable
	7	Y, B	X, A	(+,+) exp.	-	-, + , +	[3]	stable
	8	Y, A	X, B	(+,+)	-	-, + , +	-	stable
	9	X, B	Y, A	(+,+) exp.	-	-, + , +	[4]	stable
	10	X, A	Y, B	(+,+)	[5]	-, + , +	[6]	stable
	11	X, Y	A, B	(+,+) exp.		-, + , +	[7]	stable
	1	A, B	X, Y	(+, +)	-	-, +, -	[8]	stable focus
CRM_2	2	A, B	X, Y	(+, +)		+, +, -	[9]	unstable focus
(Br.)	3	A, B	X, Y	(+, +)		-, +, +	[8]	stable
	4	A, B	X, Y	(+, +)	[8]	+, +, +	[9]	unstable
	5	A, B	X, Y	(+, +)	[8]	0, +	[10]	Hopf bifurcation

Table SI 1: Type of steady state solutions and classification of critical points for CRM_1 and CRM_2 (Br.) equation systems. In the order of the columns: current number (No.), the fixed variables (Fix.), the variables of the ODEs (Var.), the solution of the ODE system (Sol.), the conditions for the solution to be the specified one (Cond.), the signs for the trace Tr(J), the determinant Det(J) and $S(J) = Tr(J)^2 - 4Det(J)$ of the ODE's Jacobian, the conditions for Tr(J), Det(J) or S(J) the be the specified one (Cond.) and the nature of the critical point. "exp." in the solution column stands for "experimental" signaling that the solutions obtained are real and positive but only for specific values given to coefficients and initial conditions. The circled notations stand for [1]: $y_{sol}, b_{sol} > 0$ if $\alpha > \beta$, [2]:$y_{sol} > 0$ for $a > 0$, [3]: $S(J) > 0$ if $4a^{1+f}(a^d d + a^g bg)y < (a^{1+f} + a^f fx + a^d dy + a^g bgy)^2$, [4]: $S(J) > 0$ if $4a^{1+f}(a^d + a^g b)fx < (a^{1+d} + a^{1+g}b + a^f fx + a^d dy + a^g bgy)^2$, [5]: $y_{sol}, b_{sol} > 0$ if $z^{1/\beta} > a^f x$, [6]: $S(J) > 0$ if $4a^{d+g}y < (a^d + a^g(b + y))^2$, [7]: $S(J) > 0$ if $4a^{1+g}y(2a^f fx + a^d dy) < (a^f fx + a^{1+g}y + a^d dy + a^g bgy)^2$, [8]: $Tr(J) < 0$ if $b < 1 + a^2$, [9]: $Tr(J) > 0$ if $b > 1 + a^2$, [10]: $Tr(J) = 0$ if $b = 1 + a^2$.
